# CircCOG8 Downregulation Contributes to the Compression-Induced Intervertebral Disk Degeneration by Targeting miR-182-5p and FOXO3

**DOI:** 10.3389/fcell.2020.581941

**Published:** 2020-10-21

**Authors:** Qian Xiang, Liang Kang, Kangcheng Zhao, Juntan Wang, Wenbin Hua, Yu Song, Xiaobo Feng, Gaocai Li, Saideng Lu, Kun Wang, Cao Yang, Yukun Zhang

**Affiliations:** Department of Orthopaedics, Union Hospital, Tongji Medical College, Huazhong University of Science and Technology, Wuhan, China

**Keywords:** nucleus pulposus cell, intervertebral disk degeneration, mechanical stress, circular RNA, circCOG8, microRNA sponge

## Abstract

Circular RNAs (circRNAs) have been increasingly demonstrated to play critical roles in the pathogenesis of various human diseases. Intervertebral disk degeneration (IDD) is recognized as the major contributor to lower back pain, and mechanical stress is a predominant trigger for IDD. However, little is known about the part that circRNAs play in the involvement of mechanical stress during IDD development. In the present study, we identified a novel circRNA and examined the role of this circRNA in a compression loading-induced IDD process. We detected the expression pattern of circCOG8 and observed its function in disk NP cells under mechanical stress. We conducted bioinformatics analysis, RNA immunoprecipitation experiment, and reporter gene assay to unveil the mechanism of the circCOG8 downregulation mediated IVD degeneration. Results showed that the circCOG8 expression was obviously down-regulated by the mechanical stress in disk NP cells. CircCOG8 attenuated NP cells apoptosis, intracellular ROS accumulation, and ECM degradation *in vitro* and *ex vivo*. CircCOG8 directly interacted with miR-182-5p and, thus, modulated the FOXO3 expression to affect the compression-induced IDD progression. Altogether, the present study revealed that the circCOG8/miR-182-5p/FOXO3 pathway was an important underlying mechanism in the involvement of compression during the IDD progression. Intervention of circCOG8 is a new therapeutic strategy for IDD treatment.

## Introduction

Lower back pain is a global health problem with a high morbidity and it is the leading cause of job disability in the modern society (Hoy et al., 2014). Although there are various initiating contributors associated with lower back pain, it is widely accepted that the intervertebral disk degeneration (IDD) is the major reason (Hughes et al., 2012). However, the pathogenesis and etiology of IDD have not been explicitly clarified yet. Physiologically, the intervertebral disk (IVD) comprises the inner gelatinous nucleus pulposus (NP), the surrounding collagenous annulus fibrosus (AF), as well as the cartilaginous endplates (EP), and the disks act as the most important load-bearing structure of the spine (Roberts et al., 2006). Disk NP cells are in the center of IVD and play a crucial part in maintaining the balance between the NP extracellular matrix (ECM) catabolism and anabolism. The abundance of ECM, including the proteoglycans and type II collagen generated by the NP cells, make important contributions to the physiological structure and function of IVD (Roughley, 2004). And, IVD degeneration is typically characterized by the aberrant conditions of NP cells that the ECM catabolism exceeds anabolism, cell apoptosis rates are abnormally increased, and reactive oxygen species (ROS) generation and antioxidant defense get out of balance to cause oxidative stress (Zhao et al., 2007; Risbud and Shapiro, 2014; Feng et al., 2017). Deep research into the underlying mechanism of these aberrant activities of the NP cells can help clinicians get a better understanding of the IDD pathogenesis and, thus, provide new strategies for the IDD treatment (Hughes et al., 2012; Sakai and Grad, 2015).

Mechanical stress is a critical etiological factor that provokes IVD degeneration (Setton and Chen, 2006; Neidlinger-Wilke et al., 2014). The pressure of the human IVD ranges from 0.1 to 1.1 MPa *in vivo* depending on the postures and the disk is also subjected to various non-physiological mechanical loading, which, in return, contributes significantly to the disk damage (Wilke et al., 1999; Adams and Roughley, 2006). Studies have reported that mechanical loads could induce the ECM degradation via shifting the balance of the ECM metabolism homeostasis toward catabolism, accompanied by an elevated expression of catabolic genes, such as disintegrin and metalloproteinase with thrombospondin motifs (ADAMTS), as well as, matrix metalloproteinases (MMP) (Yurube et al., 2012; Holguin and Silva, 2018). Meanwhile, it was demonstrated that mechanical compression contributed to excessive ROS accumulation to promote oxidative stress in NP cells (Li et al., 2017). In our previous work, we elucidated that applying a static compression of 1.0 MPa to human NP cells obviously induced programmed cell death by which it was deeply involved in disk injury (Li et al., 2018). Furthermore, we also unveiled that mechanical loading could effectively induce various pathological responses of disk cells in an *ex vivo* organ-cultured rat IVD model (Wu et al., 2019).

Accumulating evidence have revealed that non-coding RNAs, including microRNAs (miRNAs) and circular RNAs (circRNAs), functionally participate in the pathophysiological process of IDD (Li et al., 2015, 2019). MiRNAs are highly conserved, short, and single stranded endogenous RNAs which can regulate the target mRNA expression via associating with AGO proteins (Bartel, 2004). For instance, miR-7 was found to be a critical contributor to the IDD by targeting GDF5 in disk NP cells, as reported in our previous work (W. Liu et al., 2016). In the present study, results revealed that miR-182-5p contributed to the compression-induced damage in NP cells which was obviously upregulated under compression stress. CircRNAs are another type of understudied non-coding RNAs and they have a closed continuous loop which enables them to be more stable compared to the linear RNAs and resistant to degradation by exonucleases or RNase R (Han et al., 2018). In regard to the biological function of circRNAs, evidence has claimed that a number of circRNAs can sequester miRNAs to indirectly regulate the corresponding target genes expression which was also called “miRNA sponge” (Memczak et al., 2013). The most representative miRNA sponge is that the Cdr1as, an antisense transcript of cerebellar degeneration-related protein 1, harbors 74 conserved binding sites for miR-7 (Hansen et al., 2013). It has been reported that circRNAs exerted significant effects on the initiation and development of degenerative disk diseases by functioning as competing endogenous RNAs and consequently modulating target mRNAs levels (Cheng et al., 2018; Song et al., 2018). However, little is known about the part circRNAs play in the IDD progression induced by mechanical stress. Therefore, this study was aimed to explore a possible role of circRNAs in the mechanisms leading to IDD by examining their expression in disk NP cells under mechanical stress. We identified a novel circular RNA circCOG8 based on the circRNA microarray and bioinformatics analysis and systemically investigated the role of this circRNA in the progression of compression-loading-induced IDD.

## Results

### CircCOG8 Was Significantly Down Regulated in the Disk NP Cells Under Mechanical Stress

**FIGURE 1:**
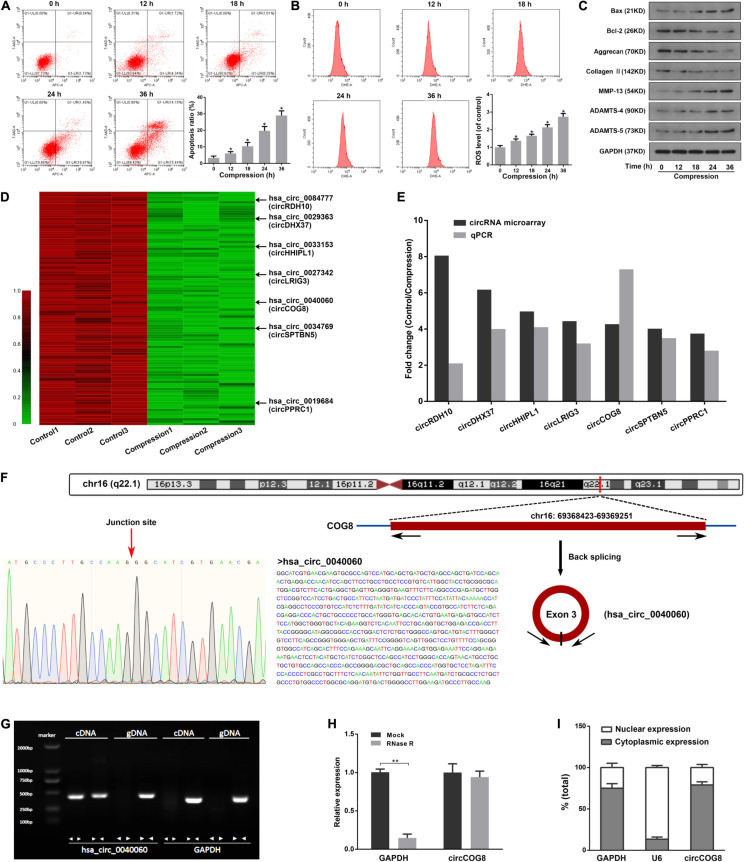
CircCOG8 was significantly down-regulated in the compression-treated human NP cells. **(A)** The human NP cells were treated with 1 MPa compression for the indicated time points (0, 12, 18, 24, and 36 h), and the cell apoptosis was determined by the Annexin V-APC and 7-AAD double-staining with subsequent flow cytometry analysis. Data were represented as mean ± SD. 0 h group served as a control, *p < 0.05 versus control, *n* = 3 (ANOVA with Tukey’s post-test). **(B)** The intracellular ROS production of the NP cells with compression for indicated time points were examined by the ROS-specific fluorescent probe DHE and measured by the flow cytometry analysis. Data were represented as mean ± SD. 0 h group served as a control, *p < 0.05 versus control, *n* = 3 (ANOVA with Tukey’s post-test). **(C)** Western blot analysis was used to detect the protein expression level of ECM anabolism markers (Aggrecan, Type II collagen), ECM catabolism enzymes (MMP-13, ADAMTS-4, ADAMTS-5), and apoptosis-associated markers (Bax, Bcl-2). Data were normalized to the GAPDH control. The GAPDH band served as a common internal loading control for Bax, Bcl-2, Aggrecan, Type II collagen, MMP-13, ADAMTS-4, and ADAMTS-5. **(D)** Clustered heat map showed the top 100 significantly down-regulated circRNAs in three samples of the compression treated human NP cells and three control samples. The arrows indicated the seven candidate circRNAs which were further validated using a real-time qPCR analysis. **(E)** Comparison of the fold changes for the seven candidate circRNAs from the circRNA array and real-time qPCR validation. **(F)** The genomic loci of the COG8 gene and circCOG8. The expression of circCOG8 was validated by the Sanger sequencing. **(G)** The products amplified using the divergent or convergent primers were verified by agarose gel electrophoresis. Divergent primers were used to amplify circCOG8 in cDNA but not in genomic DNA (gDNA). Convergent primers could amplify both the circCOG8 and linear RNA GAPDH. **(H)** The RT-qPCR analysis of circCOG8 and linear control GAPDH mRNA after treatment with RNase R in the disk NP cells. **p < 0.05, *n* = 3. **(I)** RT-qPCR assay in nuclear and cytoplasmic fractions showed the levels of nuclear control transcript (U6), cytoplasmic control transcript (GAPDH), and circCOG8.

To uncover the potential role of circRNAs in the progression of IDD induced by mechanical loading, we examined the effects of compression on human disk NP cells in three different samples. Compression treatment significantly increased the NP cell apoptosis and intracellular ROS accumulation, increased the expression of Bax, ADAMTS-4, ADAMTS-5 as well as MMP-13, and inhibited the expression of Bcl-2, Aggrecan as well as Type II collagen (Figures 1A–C). It was suggested that the compression treatment resulted in elevated cell apoptosis, ROS accumulation, and ECM degradation in human disk NP cells. Then, we performed circRNAs microarray assays using three pairs of disk cells samples treated by 1.0 MPa mechanical loading for 36 h. Data revealed that a total of 1,784 circRNAs were significantly dysregulated, among which, 1,498 gene items were downregulated by mechanical loading compared with the control. Figure 1D showed the top 100 most significantly down-regulated circRNAs. Since most of the differentially expressed circRNAs were down-regulated in the compression treatment group, we then selected seven out of the top 100 remarkably down-regulated circRNAs for the validation in three different pairs of cell samples by real-time qPCR analysis and these seven circRNAs were all rich in miRNA binding sites. The log2 fold-changes of microarray data and RT-qPCR data were both calculated and we found that, of the seven circRNAs, the circCOG8 showed the highest fold-change (Figure 1E). As a result, the circCOG8 was chosen as the subject for further experiments. CircCOG8 (hsa_circ_0040060) is generated from back-spliced exon 3 of COG8 which is located on chromosome 16q22.1 (chr16:69368423-69369251), as annotated in the circBase online database using the human reference genome GRCh37/hg19 (Figure 1F). The length of the circCOG8 is 828 nt (nucleotide). The PCR assay suggested that divergent primers could amplify the circular circCOG8 in cDNA but not in gDNA, and convergent primers could amplify both the circCOG8 and linear control GAPDH (Figure 1G). Sanger sequencing was performed to confirm the amplified products using divergent primers, as shown in Figure 1F. CircCOG8 was resistant to RNase R exonuclease digestion, further supporting the notion that this type of RNA was circular (Figure 1H). Moreover, the circCOG8 was primarily distributed in the cytoplasm, as examined by the cytoplasmic and nuclear RNA isolation followed by RT-qPCR in disk NP cells (Figure 1I).

### Overexpression of CircCOG8 Protected Disk NP Cells From Mechanical Stress-Induced Cellular Damage

**FIGURE 2:**
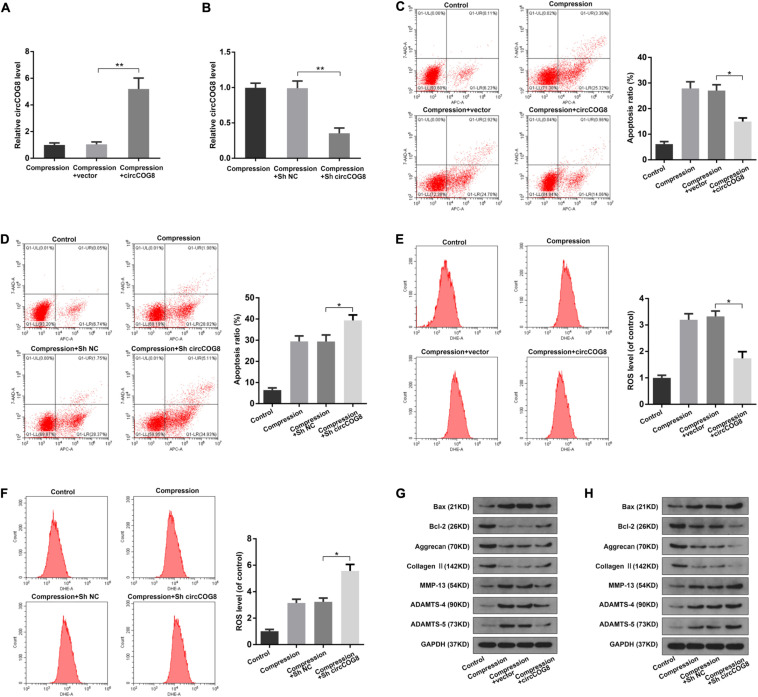
Function of circCOG8 in disk NP cells under mechanical stress. **(A,B)** The human NP cells under compression treatment were transfected with the circCOG8 overexpression vector or circCOG8 shRNA, and the circCOG8 RNA level was examined using the RT-qPCR analysis. **(C,D)** The apoptosis ratio of the NP cells transfected with the circCOG8 overexpression vector or circCOG8 shRNA was determined by the flow cytometry analysis after the Annexin V-APC and 7-AAD double-staining. **(E,F)** The intracellular ROS levels of the NP cells transfected with the circCOG8 overexpression vector or circCOG8 shRNA were detected by DHE and measured by the flow cytometry analysis. **(G,H)** Human NP cells under compression were treated with the circCOG8 overexpression or circCOG8 shRNA, and the protein levels of Bax, Bcl-2, Aggrecan, Type II collagen, MMP-13, ADAMTS-4, and ADAMTS-5 were determined by the western blot analysis. The corresponding GAPDH band served as a common internal loading control for Bax, Bcl-2, Aggrecan, Type II collagen, MMP-13, ADAMTS-4, and ADAMTS-5. Data were represented as mean ± SD. *p < 0.05, **p < 0.01, *n* = 3 (Student’s *t*-test).

Next, we transfected the disk NP cells with the circCOG8 expression adenovirus vector to overexpress circCOG8, or with shRNA targeting the junction site of circCOG8 to knockdown circCOG8. As revealed in Figure 2A, the NP cells transfected with the circCOG8 overexpression vector showed a higher circCOG8 expression level under the compression treatment. In contrast, the circCOG8 shRNA obviously resulted in the circCOG8 downregulation (Figure 2B). Then, we used flow cytometry analysis by Annexin V/PI dual staining to evaluate the circCOG8’s effect on the NP cells apoptosis under compression. We found that the circCOG8 overexpression obviously decreased the apoptosis incidence of NP cells (Figure 2C) and circCOG8 knockdown increased the apoptosis rate of NP cells under compression (Figure 2D). The intracellular ROS level were examined using the fluorescent probe DHE and flow cytometry which indicated that circCOG8 could markedly inhibit the ROS production of NP cells (Figure 2E) and the circCOG8 shRNA could promote the ROS production of disk NP cells induced by mechanical loading (Figure 2F). Western blot analysis suggested that the circCOG8 knockdown increased the protein level of ADAMTS-4, ADAMTS-5, MMP-13 as well as Bax and decreased the expression of Type II collagen, aggrecan as well as Bcl-2 while the circCOG8 overexpression partially counteracted the effects of mechanical loading on these gene expression (Figures 2G,H). The results above demonstrated that circCOG8 could alleviate the effect of mechanical stress on disk NP cells.

### CircCOG8 Functioned as miR-182-5p Sponge to Inhibit Its Expression

**FIGURE 3:**
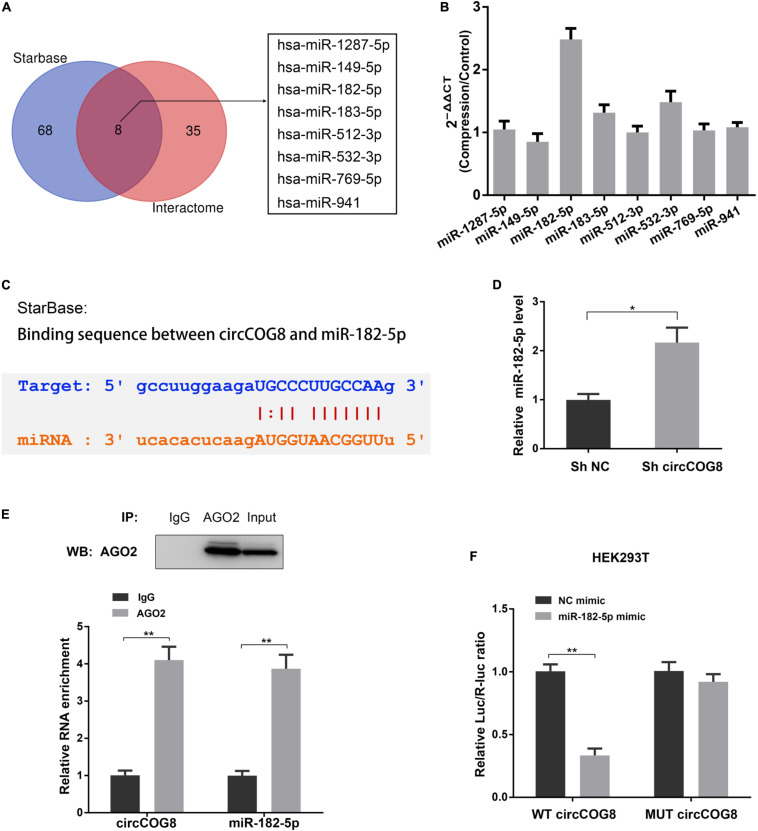
CircCOG8 acted as a sponge for miR-182-5p to inhibit its expression. **(A)** The Veen diagram revealed the eight candidate circCOG8-targeting miRNAs identified using the StarBase online tool and the Circular RNA Interactome online database. **(B)** The expression pattern of these eight candidate miRNAs in the disk NP cells under mechanical stress were determined by the real-time qPCR analysis. The upregulated magnitudes of these eight miRNAs were shown as fold changes of the compression treated cell samples to control cell samples. **(C)** Schematic of the binding sequence between circCOG8 and miR-182-5p based on the StarBase online database. **(D)** The expression level of miR-182-5p in the disk NP cells transfected with the circCOG8 shRNA or control was examined by the real-time qPCR analysis. **(E)** The RNA immunoprecipitation (RIP) assay was used to detect circCOG8 and miR-182-5p enrichment. The AGO2 protein level was examined by the IP-western blot analysis (upper) and co-precipitated circCOG8 and miR-182-5p RNA levels were examined using the qRT-PCR analysis (lower). **(F)** Wild-type (WT) or mutant (MUT) circCOG8 luciferase reporter vectors were co-transfected with the miR-182-5p mimic or NC mimic into HEK-293T cells. The relative luciferase activity was determined as the ratio of firefly luciferase/Renilla luciferase (Luc/R-luc). Data were represented as mean ± SD. *p < 0.05, **p < 0.01, *n* = 3 (Student’s *t*-test).

Considering the circular RNAs can interact with microRNAs to function as microRNA sponges, circCOG8 might target a critical microRNA to regulate its activity. Firstly, we used the StarBase online tool and Circular RNA Interactome online database to predict the targeting miRNAs of circCOG8, and found that there were eight candidate miRNAs identified by overlapping the predicting results from these two databases (Figure 3A). Then, we used real-time qPCR analysis to determine whether these predicted miRNAs were dysregulated in the disk NP cells under mechanical stress. Results showed that the miR-182-5p was the only significantly upregulated miRNA with a fold change > 2 (Figure 3B). According to the StarBase online database, there was a potential binding site between the circCOG8 and miR-182-5p seed region (Figure 3C). Next, we performed the subsequent experiments to investigate the ability of the circCOG8 to directly interact with the miR-182-5p in disk NP cells. We transfected the disk NP cells with the circCOG8 shRNA to inhibit the circCOG8 expression, with the results proving that the miR-182-5p level was obviously enhanced after the circCOG8 knockdown (Figure 3D). Then, we conducted the Argonaute-2 (AGO2) immunoprecipitation by RT-qPCR to examine whether the AGO2 was associated with the miR-182-5p transcripts which could bind circCOG8. We found that both the circCOG8 and miR-182-5p were highly enriched by the anti-AGO2 antibodies (Figure 3E). Furthermore, the pMIR-REPORT luciferase reporter plasmids with mutant (MUT) or wild type (WT) circCOG8 fragment which possessed miR-182-5p complementary binding sequence were co-transfected to the HEK293T cells with miR-182-5p. Results revealed that the relative Luc/R-luc ratio of WT luciferase reporter was obviously lower in the miR-182-5p mimic group compared to the negative control (Figure 3F). In summary, the above data demonstrated that circCOG8 functioned as an miR-182-5p sponge to suppress its expression in the disk NP cells.

### MiR-182-5p Facilitated Mechanical Stress-Induced Cellular Damage of Disk NP Cells

**FIGURE 4:**
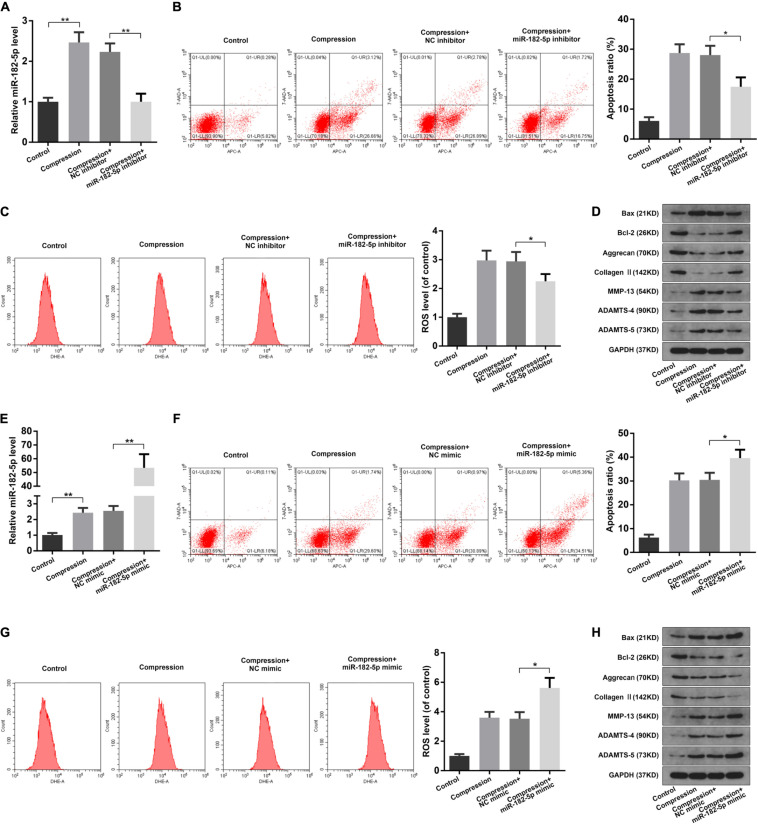
Function of miR-182-5p in disk NP cells under mechanical stress. **(A)** The human NP cells under compression were transfected with the miR-182-5p inhibitor and the miR-182-5p expression level was examined using the RT-qPCR analysis. **(B)** The apoptosis ratio of the NP cells with the miR-182-5p inhibitor treatment was evaluated using the Annexin V-APC and 7-AAD double-staining with subsequent flow cytometry analysis. **(C)** The intracellular ROS accumulation of the NP cells with the miR-182-5p inhibitor treatment were detected by DHE and measured by the flow cytometry analysis. **(D)** The protein expression levels of Bax, Bcl-2, Aggrecan, Type II collagen, MMP-13, ADAMTS-4, and ADAMTS-5 were determined by the western blot analysis. The GAPDH band served as a common internal loading control for Bax, Bcl-2, Aggrecan, Type II collagen, MMP-13, ADAMTS-4, and ADAMTS-5. **(E)** The human NP cells under compression were transfected with the miR-182-5p mimic and the miR-182-5p level was determined by the RT-qPCR analysis. **(F)** The apoptosis ratio of the NP cells with miR-182-5p mimic treatment. **(G)** The intracellular ROS levels of the NP cells transfected with the miR-182-5p mimic. **(H)** The protein levels of Bax, Bcl-2, Aggrecan, Type II collagen, MMP-13, ADAMTS-4, and ADAMTS-5 were determined by the western blot analysis. The GAPDH band served as a common internal loading control for Bax, Bcl-2, Aggrecan, Type II collagen, MMP-13, ADAMTS-4, and ADAMTS-5. Data were represented as mean ± SD. *p < 0.05, **p < 0.01, *n* = 3 (Student’s *t*-test).

Since miR-182-5p is the circCOG8-targeted microRNA, we speculated that miR-182-5p could have crucial regulating effects on the disk NP cells with compression loading. To test our hypothesis, we carried out the following experiments. Using the RT-qPCR assay, we found that miR-182-5p was elevated in the disk NP cells after the compression stimulation; the miR-182-5p expression was markedly inhibited after the miRNA inhibitor treatment and increased after the miRNA mimic treatment (Figures 4A,E). As revealed in Figures 4B–D, the miR-182-5p inhibitor markedly inhibited the up-regulation of apoptosis rate, ROS production, and ECM degradation in disk NP cells induced by mechanical stress. Conversely, these changes in NP cells induced by compression stress were enhanced when co-treated with the miR-182-5p overexpression (Figures 4F–H). The above data revealed the critical role of miR-182-5p in promoting cell apoptosis, oxidative stress, and ECM degradation in the disk NP cells under mechanical stress.

### MiR-182-5p Functioned in Disk NP Cells via Interacting With FOXO3

**FIGURE 5:**
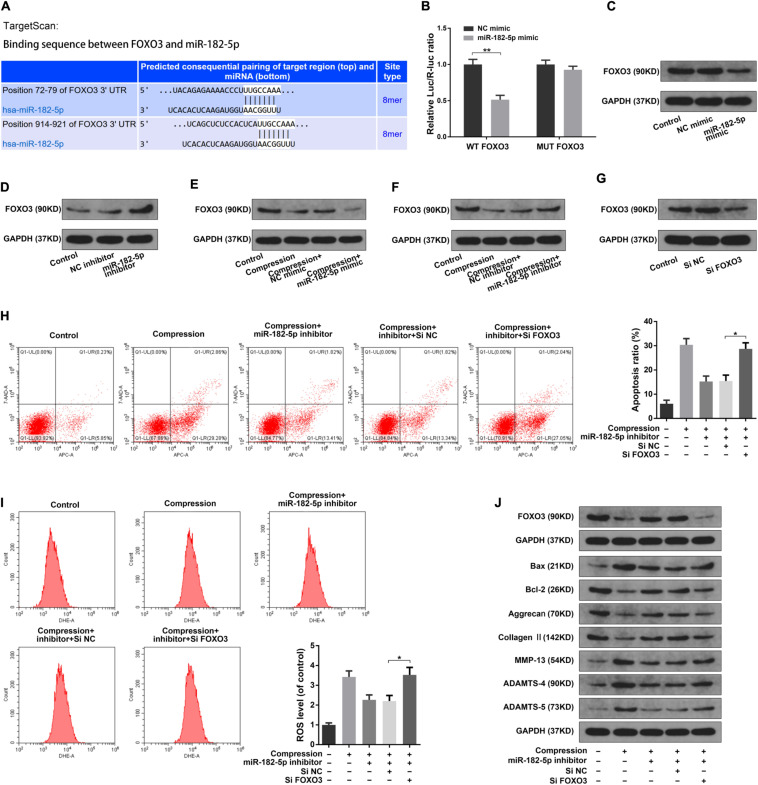
MiR-182-5p functioned in disk NP cells via targeting FOXO3. **(A)** Schematic of the binding sequence between the 3′ UTR of FOXO3 and miR-182-5p based on the TargetScan online database. **(B)** WT or MUT FOXO3 luciferase reporter vectors were co-transfected into the HEK-293T cells with the miR-182-5p mimic or NC mimic, and the luciferase reporter activity was presented as the relative Luc/R-Luc ratio. **(C,D)** The human NP cells were transfected with the miR-182-5p mimic or miR-182-5p inhibitor, the FOXO3 expression level was were determined by the western blot analysis. **(E,F)** The human NP cells under the compression treatment were transfected with the miR-182-5p mimic or miR-182-5p inhibitor, the FOXO3 expression level was were determined by the western blot analysis. **(G)** The FOXO3 protein expression level in the disk NP cells treated with the FOXO3 siRNA or siNC. **(H)** The Annexin V-APC and 7-AAD double-staining results indicating the apoptosis ratio of the NP cells. **(I)** The intracellular ROS levels in the NP cells were detected by DHE with the flow cytometry analysis. **(J)** The protein levels of FOXO3, Bax, Bcl-2, Aggrecan, Type II collagen, MMP-13, ADAMTS-4, and ADAMTS-5 were determined by the western blot analysis. The upper GAPDH band served as an internal loading control for FOXO3; the lower GAPDH band served as a common internal loading control for Bax, Bcl-2, Aggrecan, Type II collagen, MMP-13, ADAMTS-4, and ADAMTS-5. Data were represented as mean ± SD. *p < 0.05, **p < 0.01, *n* = 3 (Student’s *t*-test).

It has been reported that the transcription factors of the Forkhead box O class (FOXOs) played important regulating roles in the intervertebral disk maturation and homeostasis (Alvarez-Garcia et al., 2018; Penolazzi et al., 2018). Using the TargetScan online tool, we found that the Forkhead box O3 (FOXO3) possessed complementary sequence to the miR-182-5p seed region (Figure 5A). Then, we performed the luciferase analysis to validate that the miR-182-5p could directly target FOXO3. Luciferase reporter vector with mutant (MUT) or wild type (WT) FOXO3 3′-UTR, which possessed the miR-182-5p binding sites, were transfected to the HEK293T cells treated with miR-182-5p. As expected, miR-182-5p markedly suppress the luciferase activity of the WT-FOXO3 3′-UTR reporter while there was no significant difference in the MUT-FOXO3 3′-UTR reporter activity between the miR-182-5p mimic and control group (Figure 5B). Next, we performed the western blotting analysis in the disk NP cells to further validate the above findings. It was illustrated that miR-182-5p markedly suppressed the FOXO3 protein level (Figure 5C). Conversely, the miR-182-5p knockdown dramatically promoted the FOXO3 protein level (Figure 5D). The above results suggested that miR-182-5p could bind to FOXO3 3′-UTR to repress its expression. Then, we carried out the western blot analysis to explore the role of interaction between miR-182-5p and FOXO3 in the disk NP cells under mechanical stress. It was suggested that the level of FOXO3 protein was obviously decreased under the compression loading (Figures 5E,F). Overexpressing miR-182-5p could facilitate the inhibitive effect of mechanical stress on FOXO3 expression (Figure 5E) and the miR-182-5p inhibitor could mitigate the inhibitive effect of compression on the FOXO3 protein level (Figure 5F). Subsequently, the FOXO3 siRNA was used to further validate the critical role of FOXO3 (Figure 5G). It was revealed that the FOXO3 siRNA could partially counteract the effect of the miR-182-5p knockdown on human NP cells (Figures 5H–J). The above results demonstrated that miR-182-5p functioned in the disk NP cells through interacting with FOXO3.

### CircCOG8 Functioned in Disk NP Cells via Targeting miR-182-5p and FOXO3

**FIGURE 6:**
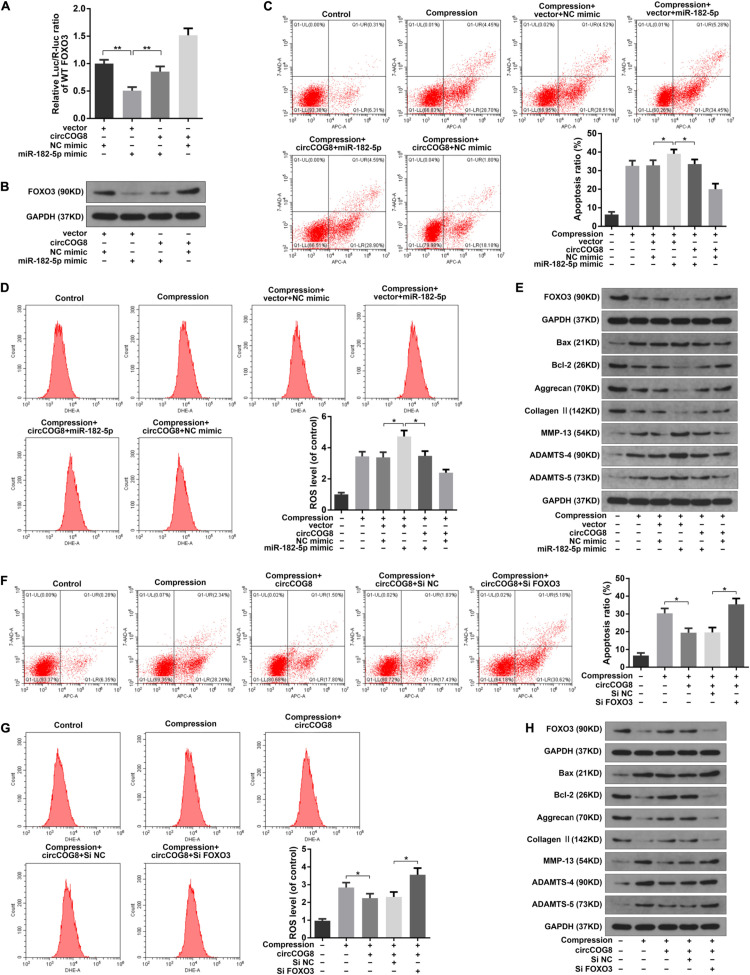
CircCOG8 functioned in the disk NP cells by targeting miR-182-5p and FOXO3. **(A)** WT FOXO3 luciferase reporter vector was co-transfected into the HEK-293T cells with the miR-182-5p mimic or NC mimic, thecircCOG8 overexpression or control vector, and the luciferase reporter activity was presented as the relative Luc/R-Luc ratio. **(B)** The FOXO3 protein expression levels of the human NP cells under compression in corresponding groups were examined by the western blot analysis. **(C)** The apoptosis ratio in the NP cells was detected by Annexin V-APC and 7-AAD double-staining. **(D)** The intracellular ROS levels in the NP cells were detected by DHE with the flow cytometry analysis. **(E)** The protein levels of FOXO3, Bax, Bcl-2, Aggrecan, Type II collagen, MMP-13, ADAMTS-4, and ADAMTS-5 were determined by the western blot analysis. The upper GAPDH band served as an internal loading control for FOXO3; the lower GAPDH band served as a common internal loading control for Bax, Bcl-2, Aggrecan, Type II collagen, MMP-13, ADAMTS-4, and ADAMTS-5. **(F)** The Annexin V-APC and 7-AAD double-staining results indicating the apoptosis ratio of the NP cells. **(G)** The intracellular ROS production in the NP cells was detected by DHE with flow cytometry analysis. **(H)** The protein levels of FOXO3, Bax, Bcl-2, Aggrecan, Type II collagen, MMP-13, ADAMTS-4, and ADAMTS-5 were examined by the western blot analysis. The upper GAPDH band served as an internal loading control for FOXO3; the lower GAPDH band served as a common internal loading control for Bax, Bcl-2, Aggrecan, Type II collagen, MMP-13, ADAMTS-4, and ADAMTS-5. Data were represented as mean ± SD. *p < 0.05, **p < 0.01, *n* = 3 (Student’s *t*-test).

To further examine the effects of circCOG8 on the FOXO3 expression with the involvement of miR-182-5p, we then carried out the following experiments. The luciferase activity assay revealed that miR-182-5p markedly repressed the WT-FOXO3 luciferase activity while circCOG8 could partially reverse this inhibitory effect (Figure 6A). Western blotting analysis also revealed that the repressive function of miR-182-5p on the FOXO3 expression level could be neutralized by the circCOG8 upregulation (Figure 6B). It was suggested that circCOG8 could function as the miR-182-5p sponge to modulate the FOXO3 expression. Subsequently, we found that the up-regulation of cell death rate, ROS production as well as the ECM catabolic activity in the disk NP cells caused by miR-182-5p could be counteracted by the circCOG8 overexpression (Figures 6C–E). Moreover, using the siRNA-mediated FOXO3 knockdown, we found that the absence of FOXO3 evidently abolished the protective effect of the circCOG8 overexpression in the disk NP cells under mechanical stress (Figures 6F–H). The above results revealed that circCOG8 exerted its functions by targeting miR-182-5p and FOXO3.

### CircCOG8 Overexpression Attenuated Compression-Induced IDD *ex vivo*

**FIGURE 7:**
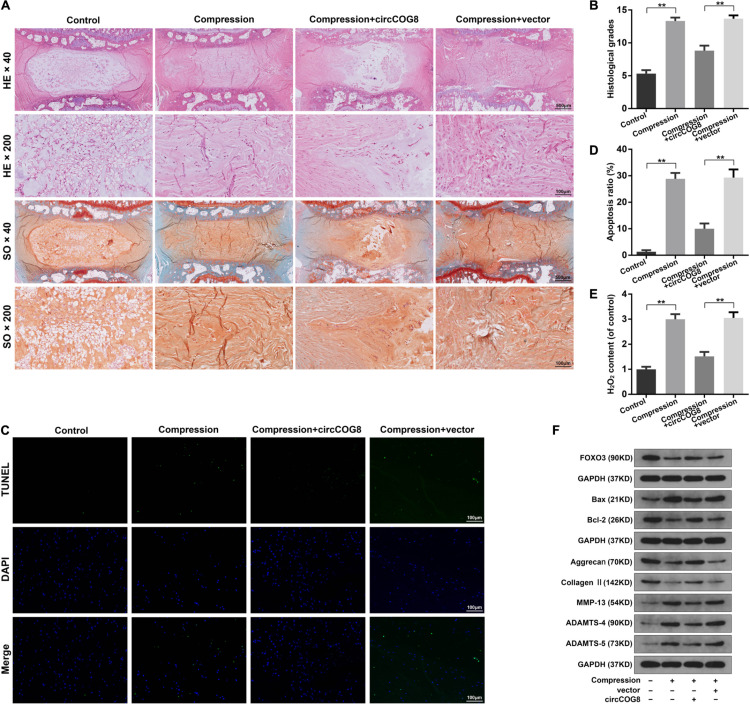
CircCOG8 overexpression attenuated the compression-induced IDD *ex vivo.* **(A)** Representative photograph of the rat IVD tissues stained with HE and SO. The images were scanned at × 40 magnification (scale bar = 500 μm) and × 200 magnification (scale bar = 100 μm). **(B)** The histological grades of the disk samples evaluated by the histological grading scale. **(C)** Representative photograph of the TUNEL analysis to examine the NP cell apoptosis (scale bar = 100 μm). **(D)** Quantitative analysis of the TUNEL-positive cells indicating the apoptosis ratio. **(E)** The H_2_O_2_ content in the IVD tissues indicating the ROS level. **(F)** Western blot analysis was used to determine the protein expression level of FOXO3, Bax, Bcl-2, Aggrecan, Type II collagen, MMP-13, ADAMTS-4, and ADAMTS-5. The upper GAPDH band served as an internal loading control for FOXO3; the middle GAPDH band served as a common internal loading control for Bax and Bcl-2; the lower GAPDH band served as a common internal loading control for Aggrecan, Type II collagen, MMP-13, ADAMTS-4, and ADAMTS-5. Data were represented as mean ± SD. *p < 0.05, **p < 0.01, *n* = 6 IVDs per group (Student’s *t*-test).

To further test the function of circCOG8 exhibited on the compression-induced IDD progression, we established the *ex vivo* organ-cultured rat disk model. The modeled rat IVDs *ex vivo* were injected with the adenovirus circCOG8 or control vector. Then, the IVD specimens were harvested and subjected to histopathological analysis (Figures 7A,B). In the control groups, the round-shaped NP tissues occupied more than half area of the disk as examined by the HE staining, and abundant proteoglycan matrix was confirmed in the NP area as indicated by the Safranin-O staining (Figure 7A). In the compression-induced IDD groups, the NP tissues area in the disk was obviously smaller with the collapsed disk height and the proteoglycan matrix volume was markedly decreased. Conversely, the circCOG8 overexpression could alleviate these disk degenerative changes induced by the compression when comparing with the IDD groups. The statistical data also suggested that the histological grades in the IDD groups were higher than that in the circCOG8 overexpression groups (Figure 7B). The TUNEL assay showed that the NP cell apoptosis ratio was elevated in the compression-induced IDD groups and markedly decreased after co-treatment of the circCOG8 overexpression (Figures 7C,D). Furthermore, compared to the IDD groups, the ROS level in IVD tissues indicated by H_2_O_2_ content was obviously decreased in the circCOG8 overexpression groups (Figure 7E). Lastly, the western blot analysis revealed that circCOG8 could alleviate the FOXO3 reduction, ECM degradation, and apoptotic response in the *ex vivo* organ-cultured model (Figure 7F). Collectively, the above results indicated that the circCOG8 overexpression could attenuate the compression-induced IDD *ex vivo*.

## Discussion

Previous studies have demonstrated that IVD degeneration was a complicated disease involving numerous pathologic processes during which the nucleus pulposus cells residing in the IVD center has played a key role (Sakai et al., 2012). The impairment of the structural and functional integrity of the NP is a classical character of IDD progression, including the imbalanced metabolism of the ECM, NP cell apoptosis, and excessive ROS accumulation (Roberts et al., 2006; Zhao et al., 2007; Feng et al., 2017). The IVD is an important load-bearing structure and it is under various degrees of mechanical stress during which the NP could counteract and distribute the mechanical loads in the spine (Wilke et al., 1999; Gilchrist et al., 2011). Accumulating evidences have corroborated that mechanical stress was a predominant initiating factor for IDD and abnormal mechanical stress induced various pathological cellular responses within the IVD (MacLean et al., 2005; Li S. et al., 2018). Recently, research demonstrated that the compression treatment on NP cells could lead to the activation of the NF-κB pathway, MAPK pathway, and the deactivation of the PI3K/Akt pathway (Jiang et al., 2018; Li et al., 2018; Glaeser et al., 2020; Huang et al., 2020), which provided new insights into the association between mechanical stress and IDD. In this study, we found that applying a static mechanical load of 1.0 MPa could induce ECM degradation, cell apoptosis, and excessive ROS production in NP cells, which was consistent with the previous findings (Li et al., 2017; Lin et al., 2017; Chen et al., 2018; Wu et al., 2019). Moreover, we also applied an *ex vivo* IDD model induced by mechanical stress in this study. Compared with the *in vitro* analysis, the *ex vivo* system provided a better model to uncover the pathogenesis of IDD, with the intervertebral disk structure and native ECM intact, which were important to further investigate the involvement of mechanical loading in IVD degeneration (Lee et al., 2006; Koerner et al., 2016; Wu et al., 2019). In the *ex vivo* model, the imbalance between the ECM deposition and degradation, the increased NP cell apoptosis, and overproduction of ROS were also detected. However, the precise molecular mechanism of the involvement of mechanical stress during IDD progression remains unclear and more efforts should be made to develop novel therapeutic targets for IDD.

Recently, evidence increasingly reported that circRNAs played crucial roles during the physiology and pathology of various human diseases (Zhao and Shen, 2017; Chen et al., 2018; Kleaveland et al., 2018; Liu et al., 2018). CircRNAs are single-stranded non-coding RNA molecules without 5′–3′ polarities and polyadenylated tails as a result of the circular structure generated by back splicing of introns, exons, or intergenic regions. With the following characteristics including the exceptional stability, conservation across species, and cell and tissue-specific expression patterns, circRNAs have been found involved in many crucial cellular processes and the dysregulation of circRNAs resulted in the initiation and development of a broad range of diseases. CircRNAs could directly bind to microRNAs and sequester them from the cytoplasm and, thus, counteract the inhibitory effect of miRNAs on their targeting mRNA (Li et al., 2018). The most representative circRNA Cdr1as possesses more than 70 miR-7-binding sites and emerging evidence indicates that the interactions between Cdr1as and its targeting miRNAs are closely associated with the pathology of some human diseases, such as neuropsychiatric disorders, melanoma invasion and metastasis, and β-cell dysfunction in diabetic conditions (Piwecka et al., 2017; Stoll et al., 2018; Hanniford et al., 2020). A previous research by Liu et al. (2017) reported that a dysregulated circular RNA named circRNA-MSR participated in the chondrocyte ECM degradation through binding to miRNAs and, thus, regulating the TNF-α expression during osteoarthritis progression. Emerging evidence has shown several upregulated or downregulated circRNAs in the degenerated disks played an important part in the IDD pathogenesis. For example, a circular RNA hsa_circ_001653 was upregulated in the degenerated disk tissues with close relation to the IVD degeneration degrees. This circRNA could inhibit the NP cell proliferation and ECM synthesis through targeting miR-486-3p to modulate CEMIP, which contributed significantly to the IDD development (Cui and Zhang, 2020). It was reported that a critical circRNA named circVMA21 was downregulated during IDD. CircVMA21 obviously inhibited inflammatory cytokines-induced disk cells apoptosis and ECM degradation by functioning as competing endogenous RNAs to bind to miR-200c and, thus, regulate the targeting gene XIAP (Cheng et al., 2018). There were also several other identified circRNAs reported to participate in the pathogenesis of IDD in the past few years (Li et al., 2019). However, it remains undetermined how circRNAs play a part functionally in the involvement of mechanical loads during the IVD degeneration development.

Although an increasing number of circRNAs have been identified across different eukaryotic species from microorganisms to animals, only a minor fraction of them have been researched for the biological implications (Conn et al., 2015). The circRNA identified in this study was a novel circRNA which played a critical role during the IDD progression involving mechanical stress. We showed that the circCOG8 expression in the disk NP cells was markedly downregulated by mechanical loading as determined by the microarray and RT-qPCR assays. CircCOG8 overexpression significantly alleviated the NP cell ECM degradation, cell apoptosis, and ROS accumulation induced by compression. And, downregulating circCOG8 could aggravate these effects of compression on the disk NP cells. Functionally, the critical microRNA miR-182-5p might mediate the effects of circCOG8 exerted on the disk cells. Using the bioinformatics prediction, the RNA immunoprecipitation assay and reporter gene analysis, we unveiled that circCOG8 could directly interact with miR-182-5p. Interestingly, miR-182-5p was found markedly upregulated in the disk NP cells under mechanical loading in this study. We showed that miR-182-5p facilitated the elevated apoptosis, oxidative stress as well as ECM degradation in the disk NP cells induced by mechanical loading. Inhibiting miR-182-5p markedly attenuated these cellular damages induced by compression loading. Generally, the regulatory function of a microRNA mainly is achieved by interacting with 3′-UTR of targeting mRNA to negatively regulate the gene expression. Using the online miRNA binding predicting tool, we found FOXO3 was an important miR-182-5p targeting gene and we further verified the interaction between them. FOXO3, known as FOXO3a or FKHR-L1, is among the Forkhead box O (FOXO) family of transcription factors which plays a crucial part in the normal physiological structure and function of IVD (Alvarez-Garcia et al., 2018). Previous study revealed that the FOXO3 expression level was obviously downregulated in severe degenerated human IVDs, contributing to the major histopathological changes during IDD progression (Penolazzi et al., 2018). In the present study, we observed that the FOXO3 expression in the NP cells was repressed by compression loading. FOXO3 was identified as the key targeting gene for miR-182-5p and their interaction accounted for a crucial part in the involvement of compression during IVD degeneration. Moreover, circCOG8 evidently neutralized the repressive function of miR-182-5p exerted on FOXO3, suggesting the role of circCOG8 as an miR-182-5p sponge to modulate the FOXO3 expression. We further validated that the absence of FOXO3 could counteract the protective effect of circCOG8 on human NP cells under compression loading by using the siRNA-mediated FOXO3 knockdown. However, the underlying mechanism by which circCOG8 and miR-182-5p could respond to mechanical stress still needed further investigation. Taken together, this study revealed that circCOG8 attenuated the mechanical stress induced damage in NP cells via restoring the FOXO3 expression achieved by sponging the miR-182-5p.

Collectively, the major findings from our study can be summarized as the following. (1) CircCOG8, derived from the third exon of COG8 gene, is a novel identified circRNA that functionally participates in the involvement of mechanical stress during IDD progression. (2) CircCOG8 can directly interact with miR-182-5p and, thus, modulate the FOXO3 expression in NP cells. (3) Compression-induced circCOG8 downregulation is an important underlying mechanism in the NP cell ECM degradation, cell apoptosis, and ROS accumulation via regulating the miR-182-5p/FOXO3 axis. In conclusion, the present study suggested that targeting the circCOG8/miR-182-5p/FOXO3 pathway provides a new therapeutic strategy for IDD treatment.

## Materials and Methods

### Patient Samples

The present study was supervised by the Clinical Research Ethics Committee of Tongji Medical College, Huazhong University of Science and Technology, and all methods were used in strict adherence with the approved guidelines. The IVD tissue specimens were obtained from patients with idiopathic scoliosis who underwent spinal surgery. Informed consents were obtained from all subjects. Patients’ medical records were collected and the IVD degeneration degrees were assessed by the Pfirrmann MRI-grading system (Pfirrmann et al., 2001). Generally, six disk tissue specimens were collected from two males and four females, aged 17–26 years old (mean: 21.3 years). The Pfirrmann grades of these disk samples were generally evaluated as Grade I or II. Among these specimens, three NP tissues were used to isolate the disk NP cells for the circRNA microarray assay and the other three were used for further *in vitro* experiments.

### Primary Cells Culture

The disk NP tissue samples were dissected and digested using 0.25% pronase for 0.5 h and 0.2% type II collagenase for 4 h at 37°C. After filtering through a 70 μm pore size mesh, the NP cells were transferred to Dulbecco’s modified Eagle medium (DMEM; Gibco, United States) with 15% fetal bovine serum (FBS; Gibco, United States), streptomycin (100 mg/ml; Gibco, United States) as well as penicillin (100 units/ml; Gibco, United States). The NP cells were cultured at 37°C in a CO_2_ incubator with a controlled humidified atmosphere composed of 95% air and 5% CO_2_. The culture medium was replaced every 3 days. The second-generation cells were used in this study. For the compression treatment, the disk NP cells were treated by a previously described compression device to provide 1 MPa static mechanical loading (Wu et al., 2019). Briefly, the stainless pressure vessel allowed for pumping compressed gas into it to form a closed high-pressure environment which was monitored with a barometer. The pressure vessel was pumped into the mixed 0.5% CO_2_ and 99.5% compressed air and the cells were placed in cell culture plates under humidified atmosphere at 37°C. Unless otherwise specified in the figures, the NP cells were treated under mechanical stress for 36 h.

### Western Blotting

Samples were lysed by a radio immunoprecipitation lysis buffer and their protein contents were determined by an Enhanced BCA Protein Assay Kit (Beyotime, China). After centrifugation, the lysates were run by sodium dodecyl sulfate-polyacrylamide gel electrophoresis gels and transferred onto the polyvinylidene fluoride membranes. Then, the membranes were blocked for 1 h at 25°C with 5% non-fat dried milk in TBST, incubated overnight at 4°C with primary antibody. The membranes were subsequently incubated with the HRP-conjugated secondary antibody (dilution 1:2,000; Abcam). Protein bands were detected with enhanced chemiluminescence reagents (Amersham). The primary antibodies used are as follows: Bax (ab32503, Abcam), Bcl-2 (ab196495, Abcam), ADAMTS-4 (ab185722, Abcam), ADAMTS-5 (ab41037, Abcam), MMP-13 (ab39012, Abcam), aggrecan (13880-1-AP, Proteintech), Type II collagen (ab34712, Abcam), and FOXO3 (ab23683, Abcam). GAPDH (#5174, Cell Signaling Technology) was used as controls.

### Flow Cytometry Analysis

The NP cell samples after various treatments were harvested and the apoptosis rates were examined by the dual-staining with Annexin V-APC and 7-AAD (KeyGen Biotech). Cell samples were washed twice by PBS and resuspended with the binding buffer then doubly stained with annexin-V and 7-AAD. And, the intracellular ROS production were examined by an ROS-specific fluorescent probe dihydroethidium (DHE, Beyotime). The stained cells from each experiment were analyzed by the FACSCalibur flow cytometer (Becton Dickinson).

### RNA Extraction and qPCR Assay

Total cellular RNA extraction was performed with the TRIzol method following the merchant guide. The NE-PER Nuclear and Cytoplasmic Extraction Reagents (Thermo Scientific) were applied to isolate the nuclear and cytoplasmic fractions, following manufacturer’s protocols. The first-strand cDNA was obtained from total RNA via a PrimeScript RT reagent kit (Takara Bio). The qPCR was carried out using SYBR Green Supermix (BioRad) and All-in-One miRNA RT-qPCR Kit (GeneCopoeia, Inc.). GAPDH was used as the internal control for mRNA and circRNA, and U6 was used to normalize miRNA level. Primers used in this study are as follows: *circCOG8* F, TTCAATGATCTGCGCCTCTG; *circCOG8* R, TTGGTCCTCAGTTGCTGGAT; *GAPDH* F, GTCTTCACCACCATGGAGAA; *GAPDH* R, TAAGC AGTTGGTGGTGCAG; *U6* F, CTCGCTTCGGCAGCACA; *U6* R, AACGCTTCACGAATTTGCGT; *MiR-182-5p stem-loop primer*, GTCGTATCCAGTGCAGGGTC CGAGGTATTCGCACTGGATACGACAGTGTG. *Divergent-circCOG8* F, ATGCTCATCTCGGCTCCAG; *Divergent-circCOG8* R, AGGAATGGCAGTCAGGATGG; *Convergent-circCOG8* F, CCATCCTGACTGCCATTCCT; *Convergent-circCOG8* R, CTGGAGCCGAGATGAGCAT; *Divergent-GAPDH* F, CACTTTGTCAAGCTCATTTCC; *Divergent-GAPDH* R, TTGGCAGTGGGGACACGGAAG; *Convergent-GAPDH* F, TCCATGCCATCACTGCCAC; and *Convergent-GAPDH* R, GTGGTCGTTGAGGGCAATG.

### Circular RNA Microarray and Bioinformatics Analysis

Total cellular RNA from mechanical loading treated the disk NP cells or corresponding control cells was isolated to carry out the microarray assay. And, the circRNAs microarray assay was performed by the CapitalBio Corporation (Beijing, China) according to standard procedures. The GeneSpring GX software was used to analyze the microarray data. The thresholds were set as follows: fold change > 2, *P*-value < 0.05 evaluated using the Student’s *t*-test. Clustered heat map was made to present the significantly down-regulated gene items in mechanical loading treated disk cells. The targeting miRNA of circCOG8 was predicted using the StarBase online database^1^ and the Circular RNA Interactome online database^2^. And, the targeting genes of miR-182-5p were predicted based on the TargetScan online tool^3^ as well as the miRDB^4^. Microarray raw data used in this study were available from the corresponding author upon reasonable request.

### RNase R Treatment

Total cellular RNA (2 μg) extracted from the human disk NP cells was treated with 3 unit/μg of RNase R and the samples were incubated for 0.5 h at 37°C. GAPDH mRNA was used as the linear control group. Three independent experiments were performed with triplicate samples.

### Luciferase Reporter Assay

The HEK-293T cells were cultured in 24-well plates to 60% confluence. Then, the cells were co-transfected with a luciferase reporter construct and miR-182-5p overexpression construct. The corresponding reporter construct of the WT pMIR-REPORT-circCOG8 as well as the MUT pMIR-REPORT-circCOG8 and the WT pMIR-REPORT-FOXO3-3′ UTR as well as the MUT pMIR-REPORT-FOXO3-3’UTR were obtained from the Obio Technology, Corp. 48 h after the transfection by lipofectamine 3000, the cells were assayed with the dual-luciferase reporter assay system (Promega), and measurements were performed on the Beckman–Coulter DTX880. Relative luciferase activity was presented as the Luc/R-Luc ratio by dividing the firefly luciferase (Luc) value by the *Renilla* luciferase (R-Luc) value.

### RNA-Binding Protein Immunoprecipitation (RIP)

The RIP analysis was carried out by using a Magna RIP RNA-Binding Protein Immunoprecipitation Kit (EMD Millipore Corporation, Billerica, MA, United States) combined with the human anti-AGO2 antibody (#2897, Cell signaling), according to the merchant guide. Briefly, after pelleting and re-suspending by RIP lysis buffer, the cells extract was treated with the RIP buffer containing A/G magnetic beads conjugated with an anti-AGO2 antibody or corresponding control IgG. Co-precipitated circCOG8, as well as miR-182-5p, was subsequently examined using the RT-qPCR assay.

### Cell Transfection

For the circCOG8 overexpression, exon 3 of human COG8 gene (828 bp) with approximate 1 kb flanking introns containing complementary Alu elements were amplified to construct the recombinant adenovirus vector (Obio Technology, Shanghai, China), as described previously (Zhang et al., 2014). For the lentiviral circCOG8 shRNA construction, oligonucleotides with the circCOG8 targeting sequences were inserted to the pLKD-CMV-G&PR-U6-shRNA vector (Obio Technology, Shanghai, China). Targeting sequence for circCOG8: CCAAGGGCATCGTGAACGA, negative control sequence: TTCTCCGAACGTGTCACGT. MiR-182-5p mimic, miR-182-5p inhibitor, as well as their corresponding negative controls, were produced by the GenePharma, Corp. FOXO3 siRNA (targeting sequence: GCATGTTCAATGGGAGCTTGGA) and its control siRNA was purchased from the Obio Technology, Corp. The NP cells were cultured with six-well plates to 70–80% confluence and then, the cell transfection was achieved by the Lipofectamine 3000 transfection reagent (Invitrogen, United States), according to the merchant guide.

### IVD Organ Model Culture

Rat IVDs of Co6/7 and Co7/8 were collected from Sprague-Dawley rats of 3 months, with an approval from the Animal Experimentation Committee of Huazhong University of Science and Technology. Interact disks were meticulously isolated and cultured by the DMEM (Gibco, United States) with 15% FBS (Gibco, United States), streptomycin (100 mg/ml; Gibco, United States) as well as penicillin (100 units/ml; Gibco, United States), according to our previous study (Wu et al., 2019). The IVDs were cultured at 37°C and the medium was replaced every 3 days. For the compression treatment, the IVD organ model was placed on the culture plates in the compression device which could provide 1 MPa static compression, as described above. According to the classic air-pressure principle, the custom-made compression apparatus was applied, as previously described, to expose the IVDs to static high pressure (Hutton et al., 1999; Wu et al., 2019). And, the IVDs were treated under continuous mechanical loading except for the time when the culture medium was replaced.

### CircRNA Injection Into *ex vivo* Cultured IVD Models

The adeno-associated virus (AAV) vector overexpressing circCOG8 and the control vector were constructed and packaged by the Obio Technology (Shanghai, China). The *ex vivo* IVD organs were randomly divided into four groups: control group (*n* = 6), compression group (*n* = 6), circCOG8 injection with compression group (*n* = 6), and control vector injection with compression group (*n* = 6). The *ex vivo* disks were injected with a 2 μl solution containing the experimental virus vector overexpressing circCOG8 or the control virus vector by using the 33-G needle. Then, the cultured IVDs were treated under a static mechanical loading for 4 weeks.

### Histological Evaluation, TUNEL Staining, and ROS Measurement in *ex vivo* Cultured IVD Models

The rat disk tissues were collected and rinsed using PBS. Disk samples were fixed by formalin and decalcified using EDTA. Then, the disks were embedded in paraffin, sectioned, and stained using the hematoxylin-eosin, as well as the safranin O. Histological evaluation grades were determined according to a previous study (Mao et al., 2011). The *in situ* apoptotic activity was examined by the TUNEL Apoptosis Assay Kit (C1088, Beyotime, China). The specimens were overlaid by the Vectashield Hard Set mounting medium containing DAPI. TUNEL positive cells were counted from three different fields of disk NP and the ratio of positive cells relative to total was determined. The ROS level in IVD tissues was measured by the Hydrogen Peroxide assay kit (NJJCbio, Nanjing, China), following the merchant guide.

### Statistical Analysis

The data were expressed as a mean value ± standard deviation (SD). Each experiment was repeated independently for three times, unless otherwise indicated. All data in the study were processed by the GraphPad Prism 7.0 software. Statistical significance was analyzed using the Student’s *t*-test for two-group comparisons, one-way analysis of variance (ANOVA) with Tukey’s post-test for multiple group comparisons. Significance levels were set as ^∗^*p* < 0.05, ^∗∗^*p* < 0.01.

## Data Availability Statement

All datasets generated for this study are included in the article/Supplementary Material.

## Ethics Statement

The animal study was reviewed and approved by the Animal Experimentation Committee of Huazhong University of Science and Technology.

## Author Contributions

QX and LK designed the study protocol. QX, LK, KZ, JW, WH, YS, XF, and SL performed the experiments. KZ, GL, and KW collected and analyzed data. QX, LK, and KZ wrote the manuscript. CY and YZ reviewed and revised the manuscript. YZ supported and supervised the study. All authors contributed to the article and approved the submitted version.

## Conflict of Interest

The authors declare that the research was conducted in the absence of any commercial or financial relationships that could be construed as a potential conflict of interest.

## Footnotes

1 http://starbase.sysu.edu.cn

2 http://circinteractome.nia.nih.gov

3 http://www.targetscan.org

4 http://www.mirdb.org
