# Supplementary material for: CircCOG8 Downregulation Contributes to the Compression-Induced Intervertebral Disk Degeneration by Targeting miR-182-5p and FOXO3
Source: Front Cell Dev Biol. 2020 Oct 21;8:581941. doi: 10.3389/fcell.2020.581941 (PMC7609857; doi:10.3389/fcell.2020.581941)
Supplement: Supplementary file 1 [file Data_Sheet_1.PDF]

Supplementary Figures

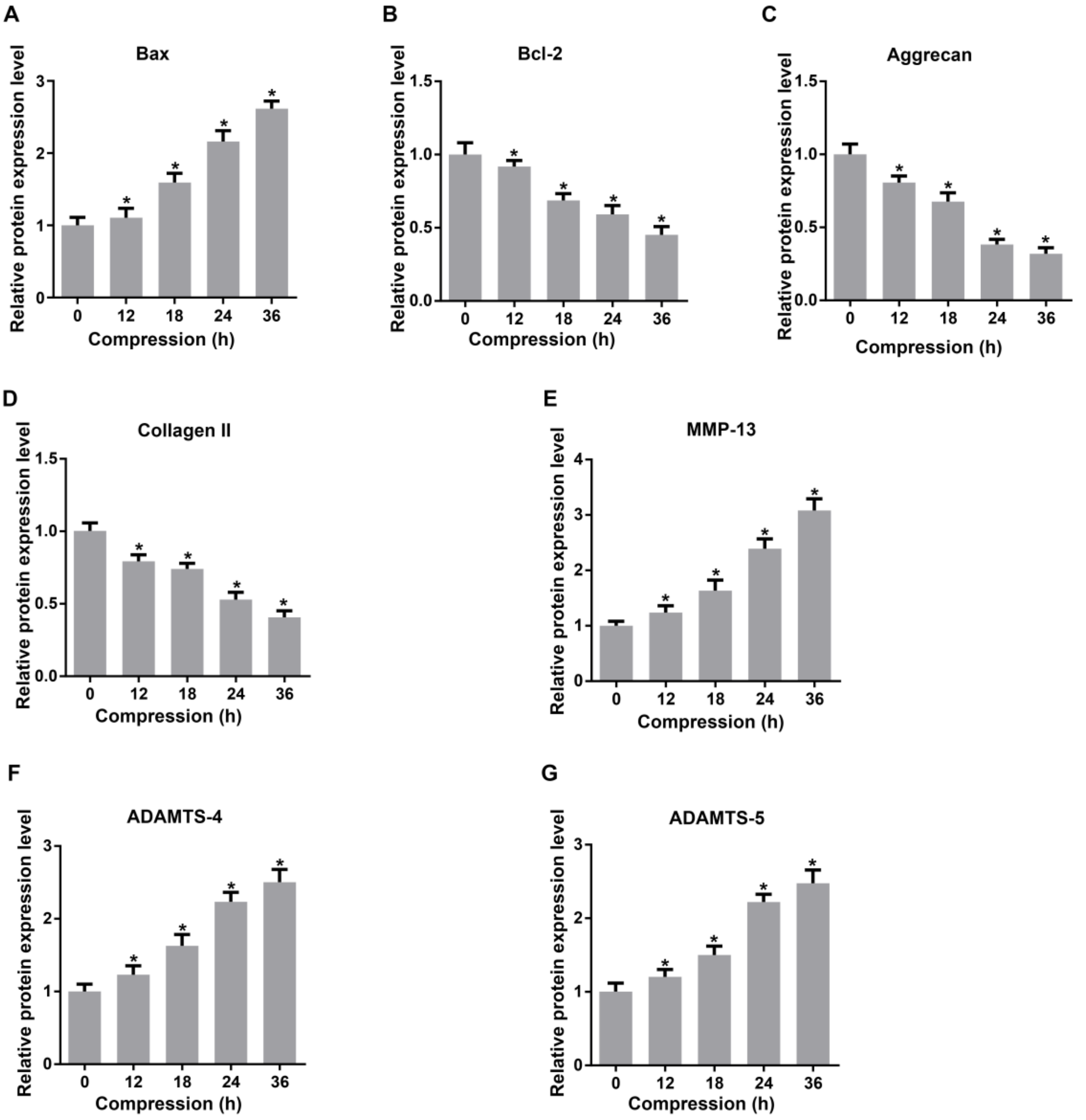

Supplementary Figure 1 | Effects of mechanical stress on human disc NP cells (corresponded to Figure 1C). **(A-G)** Quantification of apoptosis-associated proteins (Bax, Bcl-2), ECM anabolism markers (Aggrecan, Type II collagen), ECM catabolism enzymes (MMP-13, ADAMTS-4, ADAMTS-5), detected by western blot analysis. Data were represented as mean ± SD. 0 h group served as a control, $^{*}p < 0.05$, n=3 (ANOVA with Tukey's post-test).

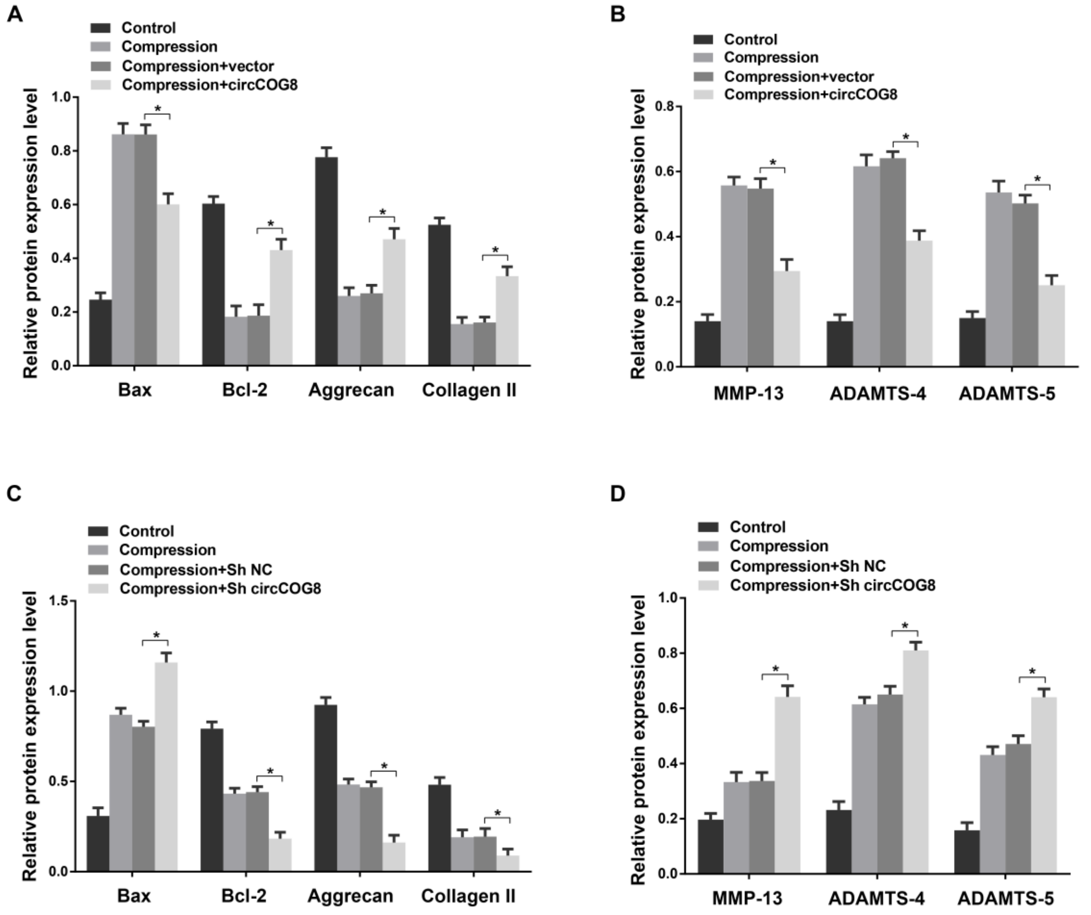

Supplementary Figure 2 | Function of circCOG8 in disc NP cells under mechanical stress (corresponded to Figure 2G-H). **(A-D)** Quantification of Bax, Bcl-2, Aggrecan, Type II collagen, MMP-13, ADAMTS-4 and ADAMTS-5, detected by western blot analysis. Data were represented as mean ± SD. $^{*}p < 0.05$, $^{**}p < 0.01$, n=3 (Student's t test).

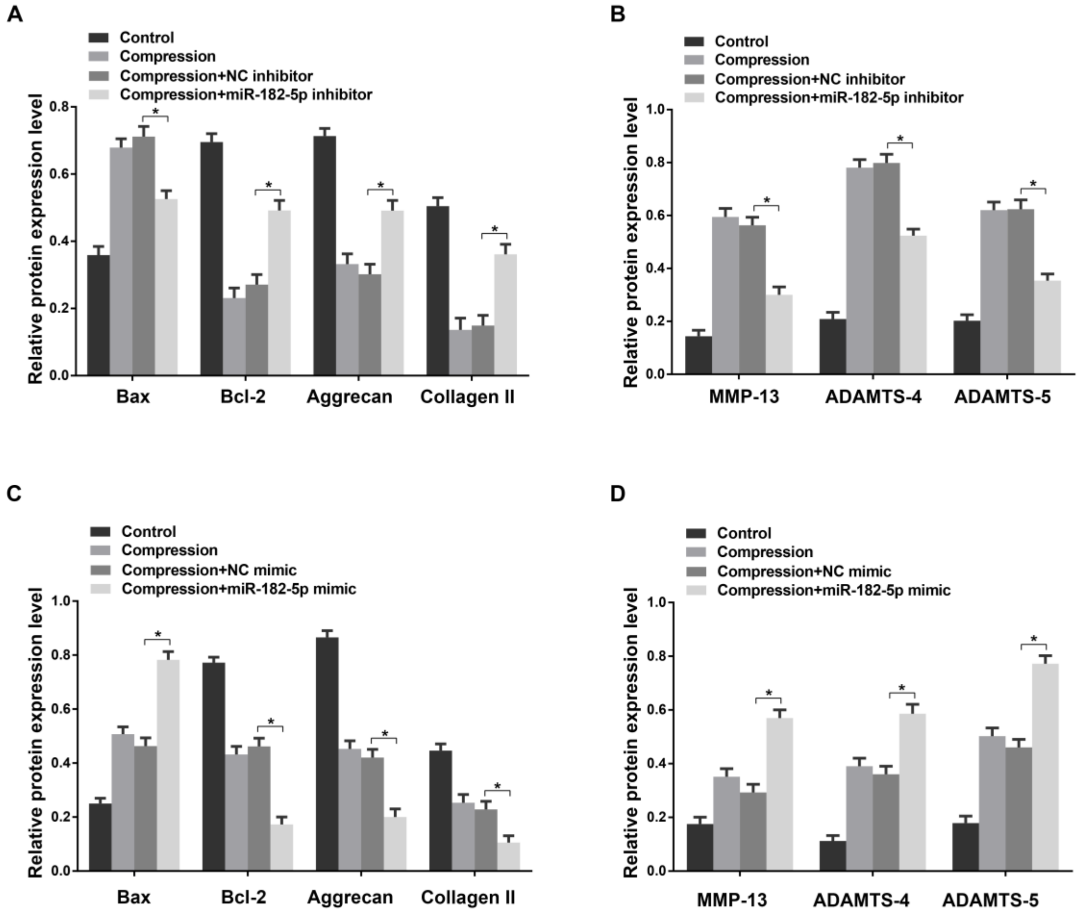

Supplementary Figure 3 | Function of miR-182-5p in disc NP cells under mechanical stress (corresponded to Figure 4D and H). **(A-D)** Quantification of Bax, Bcl-2, Aggrecan, Type II collagen, MMP-13, ADAMTS-4 and ADAMTS-5, detected by western blot analysis. Data were represented as mean ± SD. $^{*}p < 0.05$, $^{**}p < 0.01$, n=3 (Student's t test).

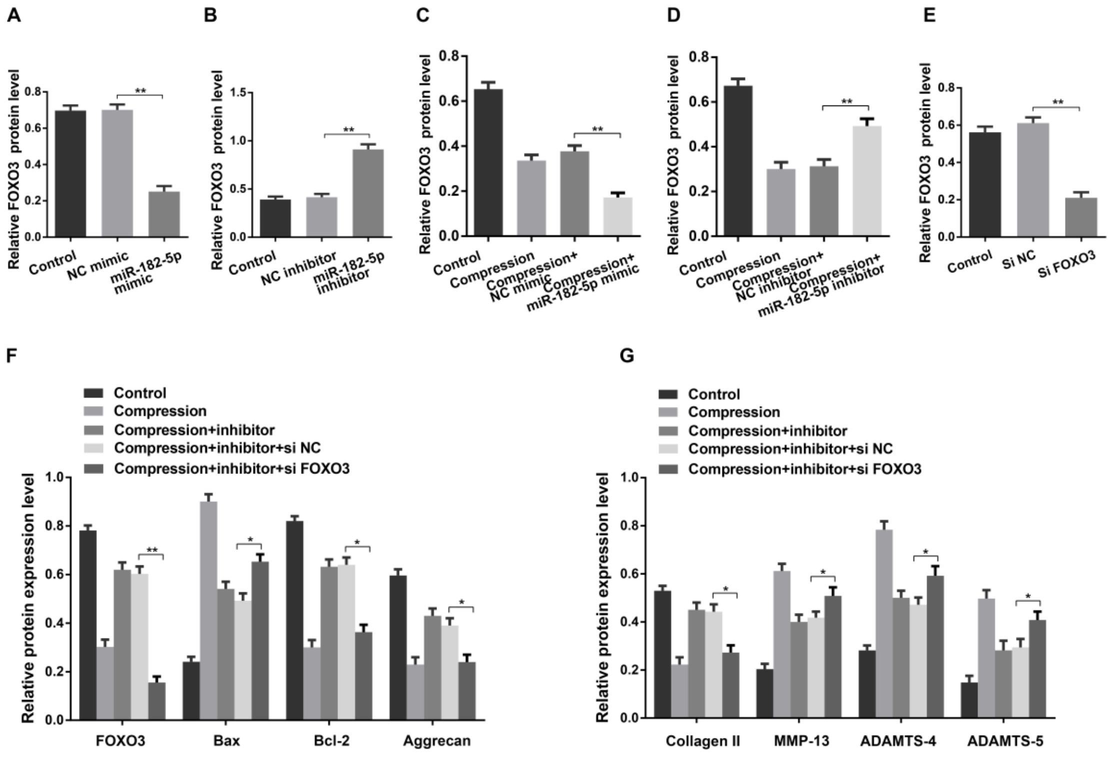

Supplementary Figure 4 | MiR-182-5p functioned in disc NP cells via targeting FOXO3 (corresponded to Figure 5C-G and 5J). **(A-E)** Quantification of FOXO3 protein expression level, detected by western blot analysis. Data were represented as mean ± SD. $^{*}p < 0.05$, $^{**}p < 0.01$, n=3 (Student's t test). **(F-G)** Quantification of FOXO3, Bax, Bcl-2, Aggrecan, Type II collagen, MMP-13, ADAMTS-4 and ADAMTS-5, detected by western blot analysis. Data were represented as mean ± SD. $^{*}p < 0.05$, $^{**}p < 0.01$, n=3 (Student's t test).

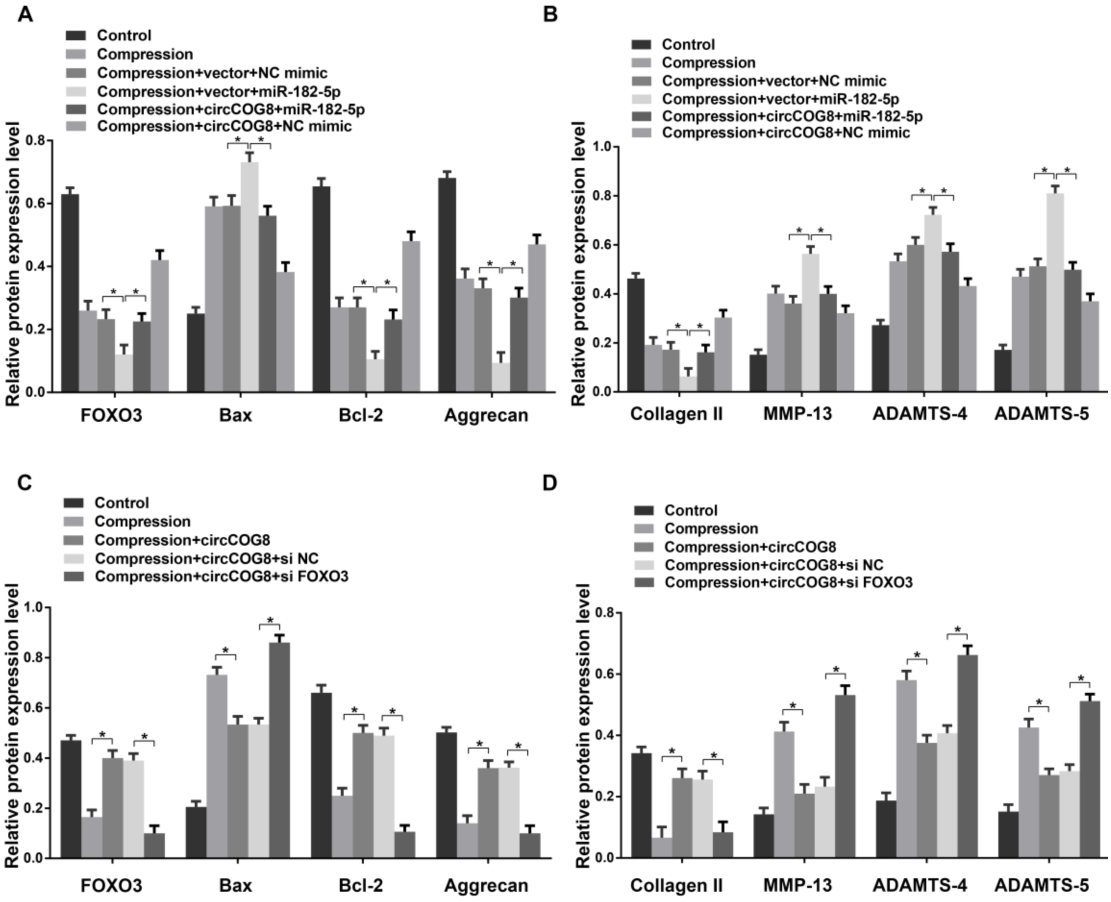

Supplementary Figure 5 | CircCOG8 functioned in disc NP cells by targeting miR-182-5p and FOXO3 (corresponded to Figure 6E and H). **(A-D)** Quantification of FOXO3, Bax, Bcl-2, Aggrecan, Type II collagen, MMP-13, ADAMTS-4 and ADAMTS-5, detected by western blot analysis. Data were represented as mean ± SD. $^{*}p < 0.05$, $^{**}p < 0.01$, n=3 (Student's t test).

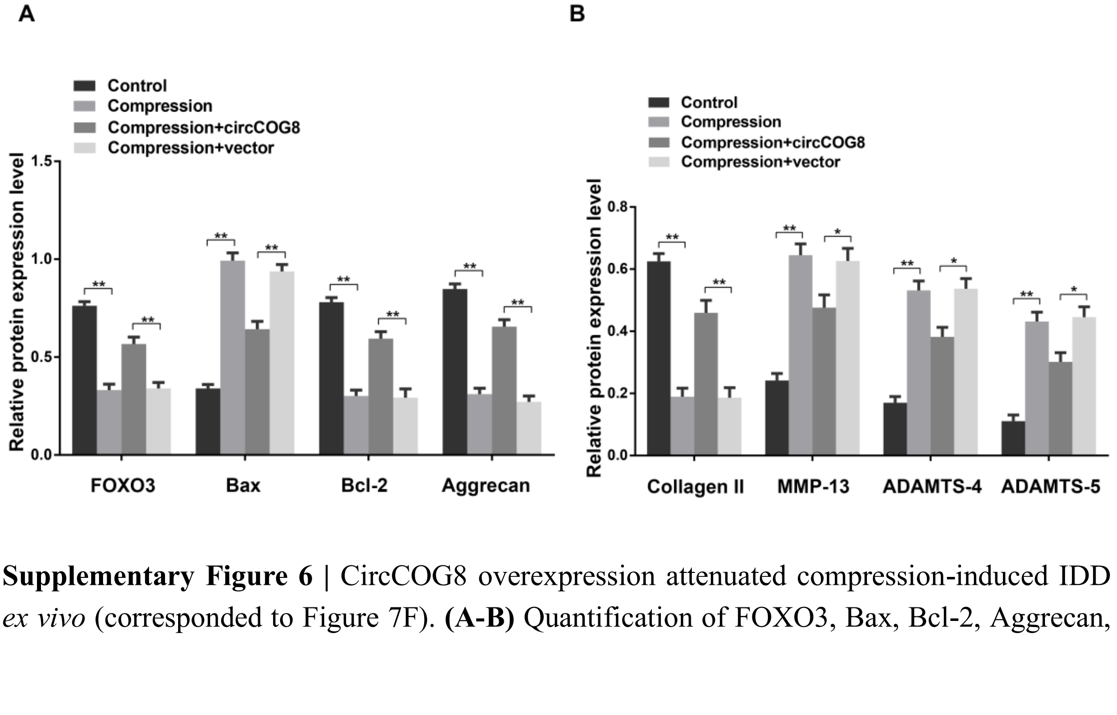

Supplementary Figure 6 | CircCOG8 overexpression attenuated compression-induced IDD *ex vivo* (corresponded to Figure 7F). **(A-B)** Quantification of FOXO3, Bax, Bcl-2, Aggrecan,

Type II collagen, MMP-13, ADAMTS-4 and ADAMTS-5, detected by western blot analysis. Data were represented as mean ± SD. $^{*}p < 0.05$, $^{**}p < 0.01$, n = 6 IVDs per group (Student's t test).
